# Physiological Consequences of Repeated Exposures to Conditioned Fear

**DOI:** 10.3390/bs2020057

**Published:** 2012-05-18

**Authors:** Robert S. Thompson, Paul V. Strong, Monika Fleshner

**Affiliations:** 1 Department of Integrative Physiology, University of Colorado at Boulder, Boulder, CO 80309-0354, USA; E-Mails: robert.s.thompson@colorado.edu (R.S.T.); paul.strong@colorado.edu (P.V.S.); 2 Center for Neuroscience, University of Colorado at Boulder, Boulder, CO 80309-0354, USA

**Keywords:** stress, diurnal rhythms, blood pressure, stress-induced hyperthermia

## Abstract

Activation of the stress response evokes a cascade of physiological reactions that may be detrimental when repeated or chronic, and when triggered after exposure to psychological/emotional stressors. Investigation of the physiological mechanisms responsible for the health damaging effects requires animal paradigms that repeatedly evoke a response to psychological/emotional stressors. To this end, adult male Sprague Dawley rats were repeatedly exposed (2X per day for 20 days) to a context that they were conditioned to fear (conditioned fear test, CFT). Repeated exposure to CFT produced body weight loss, adrenal hypertrophy, thymic involution, and basal corticosterone elevation. *In vivo* biotelemetry measures revealed that CFT evokes sympathetic nervous system driven increases in heart rate (HR), mean arterial pressure (MAP), and core body temperature. Extinction of behavioral (freezing) and physiological responses to CFT was prevented using minimal reinstatement footshock. MAP responses to the CFT did not diminish across 20 days of exposure. In contrast, HR and cardiac contractility responses declined by day 15, suggesting a shift toward vascular-dominated MAP (a pre-clinical marker of CV dysfunction). Flattened diurnal rhythms, common to stress-related mood/anxiety disorders, were found for most physiological measures. Thus, repeated CFT produces adaptations indicative of the health damaging effects of psychological/emotional stress.

## 1. Introduction

Exposure to stressors can trigger fight or flight behavioral responses and dynamic physiological changes that are highly adaptive and function together to optimize the organism’s chances of survival. The classical physiological changes that comprise the stress response include increased heart rate and cardiac contractility (QA—see methods), increased blood pressure via systemic vasoconstriction, elevated respiratory capacity, glucocorticoid evoked energy mobilization, stress-induced hyperthermia, preferential shunting of blood toward skeletal muscles and away from the digestive system, dilated pupils, and sweating in anticipation of increased physical activity [1]. Many of these dynamic changes are mediated by the sympathetic nervous system (SNS), which prepares the body for emergencies or stressors including physical threat. Interestingly, this powerful physiological cascade can be triggered after exposure to both psychological/emotional stressors [2,3,4] where physical threat is less eminent, and physical stressors where fight or flight behavioral responses can be essential for survival.

Although the stress response is beneficial when acute, it may become detrimental to many aspects of health when repeated or chronic, or when the response is triggered after exposure to emotional stressors. For example, exposure to chronic or repeated psychological/emotional stressors is a risk factor for developing and increasing the severity of cardiovascular (CV) diseases [5,6,7,8] including coronary artery disease [9,10,11,12], and for producing CV adaptations that may be preclinical indices of pathology, including changes in heart rate variability [13], ventricular arrhythmias [14,15], and vascular dominated blood pressure [16,17,18,19]. In addition to CV disturbances, chronic or repeated exposures to psychological/emotional stressors can disturb immune function [20,21,22,23], produce endocrine abnormalities [24,25] and disrupt circadian and diurnal physiological rhythms [26,27,28,29]. In fact, recent evidence suggests that stress-associated changes in circadian physiology may be etiologically linked to several adverse health consequences of repeated or chronic stress including metabolic syndrome [30,31], CV disease [32,33] and mood disorders [34,35,36].

Investigation of the mechanisms for the negative health consequences of repeated or chronic psychological stress requires an animal model that can evoke a stress response in response to a psychological based stressor. The emotional stressor that was tested in the current studies was the contextual conditioned fear test (CFT). During CFT, rodents are exposed to a shuttlebox environment (conditioned stimulus) that is paired with footshock (unconditioned stimulus). Later, when re-exposed to the shuttlebox environment, rodents demonstrate fear behavior in the absence of footshock [37,38,39]. During CFT, while rodents demonstrate fear behavior, there are increases in blood pressure and heart rate that are prevented by sympathetic ganglion blockade [40,41,42]. CFT also evokes a reliable increase in core body temperature [43,44].

The following study exposed rats to CFT at unpredictable times twice per day (inactive cycle) for 20 days. We chose this design for two reasons. First, unpredictable stressors are more aversive than predictable stressors and are thought to better mimic exposure to the variety of stressors encountered in daily life [45]. Second, stressor exposure during the inactive cycle may sleep deprive rats, which is common in humans during times of emotional stress [46] and is known to activate the SNS [47]. *In vivo* biotelemetry was used in this study because it is the gold standard for measuring labile stress-responsive changes in physiological responses such as blood pressure, heart rate, and core body temperature, and continuous data can be collected without disturbing the animals [15]. Although there are examples in the literature of biotelemetric measurement of stress-evoked changes in activity, heart rate and/or core body temperature [19,41,42,44,48,49,50,51], there are fewer examples that assess the impact of repeated psychological/emotional stressor, measure blood pressure (MAP, DBP, SBP) and cardiac contractility in addition to activity heart rate and core body temperature, and collect moment-to-moment biotelemetric measures both during and after repeated exposures to the stressor [15].

The advantages of our experimental approach are that (1) during CFT animals respond with a behavioral fear response that is a quantifiable variable; (2) the CFT is sufficient to evoke CV changes; (3) extinction of the conditioned fear response is prevented by minimal re-pairings of the shuttlebox environment and footshock; (4) biotelemetry accurately assesses real-time physiological responses in freely moving animals; and (5) both behavior (fear) and physiological responses (heart rate, blood pressure, core body temperature, spontaneous activity) can be measured simultaneously during stressor exposure, an important advance for this type of study and is clearly lacking in the literature [52]. For these reasons, repeated exposure to the CFT is a powerful addition to existing stress protocols, such as chronic variable stress [53] and repeated restraint stress [54,55], and is optimal to investigate the potentially negative health consequences of repeated exposure to emotional stressors. 

The following studies, therefore, investigated if repeated exposure to CFT (a) evoked repeatable/sustainable CV and core body temperature increases across multiple exposures; (b) produced SNS-mediated CV and core body temperature responses; and (c) produced markers indicative of pathophysiology. If repeated exposure to CFT is sufficient to evoke negative physiological consequences, then CFT exposed rats will suffer decreased body weight, increased basal corticosterone, adrenal hypertrophy, thymic involution, disturbed CV dynamics, and disrupted circadian/diurnal cycles. 

## 2. Results and Discussion

### 2.1. Experiment 1: Physiological Indices of Chronic Stress

**Figure 1:**
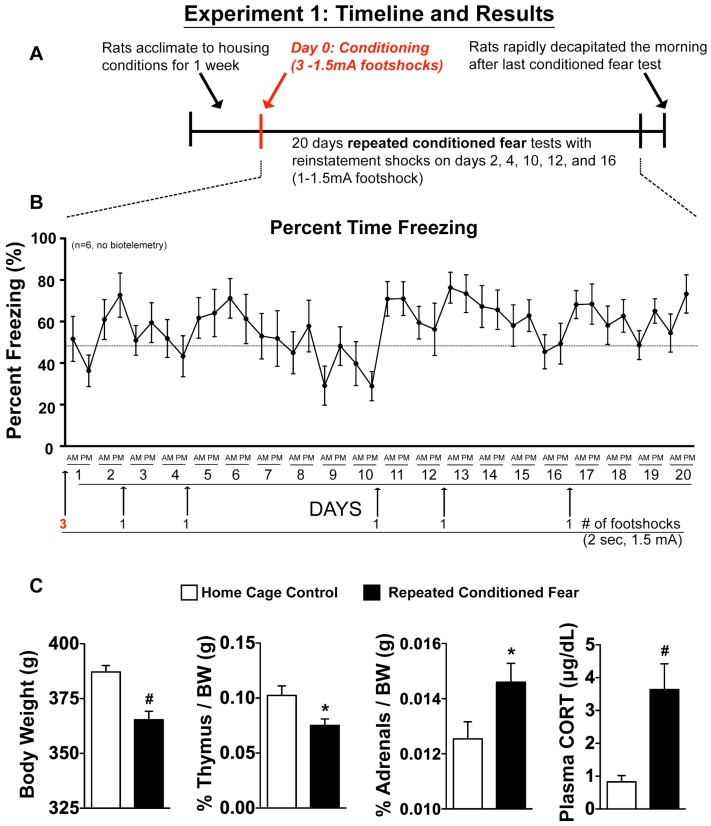
Repeated exposure to CFT with minimal reinstatement produces consistent fear behavior and evidence of chronic stress. Figure 1 depicts the timeline for experiment 1. Rats either remained in their home cage or were repeatedly exposed to CFT. Graphs show the (**A**) timeline and (**B**) the behavioral freezing response to conditioned context during 20 days repeated exposure to the CFT; (**C**) Body and thymus weights were significantly reduced as a consequence of repeated exposure to CFT, while adrenal weight and plasma CORT were increased. Abbreviations are as follows: body weight (BW), conditioned fear test (CFT), corticosterone (CORT). (* p < 0.05, # p < 0.01 compared to home cage control).

Pre-foot shock baseline levels of freezing ranged between 0–5% (data not shown). We prevented fear extinction with reinstatement footshocks and maintained animals above 50% freezing for most of the duration of repeated CFT (Figure 1B). Repeated CFT (Figure 1C) suppressed body weight gain (F_(1, 12)_ = 20.692; p = 0.0007), reduced thymus weight (F_(1, 12)_ = 5.751; p = 0.0336), increased adrenal weight (F_(1, 12)_ = 4.840; p = 0.0481), and increased basal corticosterone (F_(1, 12)_ = 15.949; p = 0.0018).

### 2.2. Experiment 2: Prazosin and Propranolol Administration during CFT

**Figure 2:**
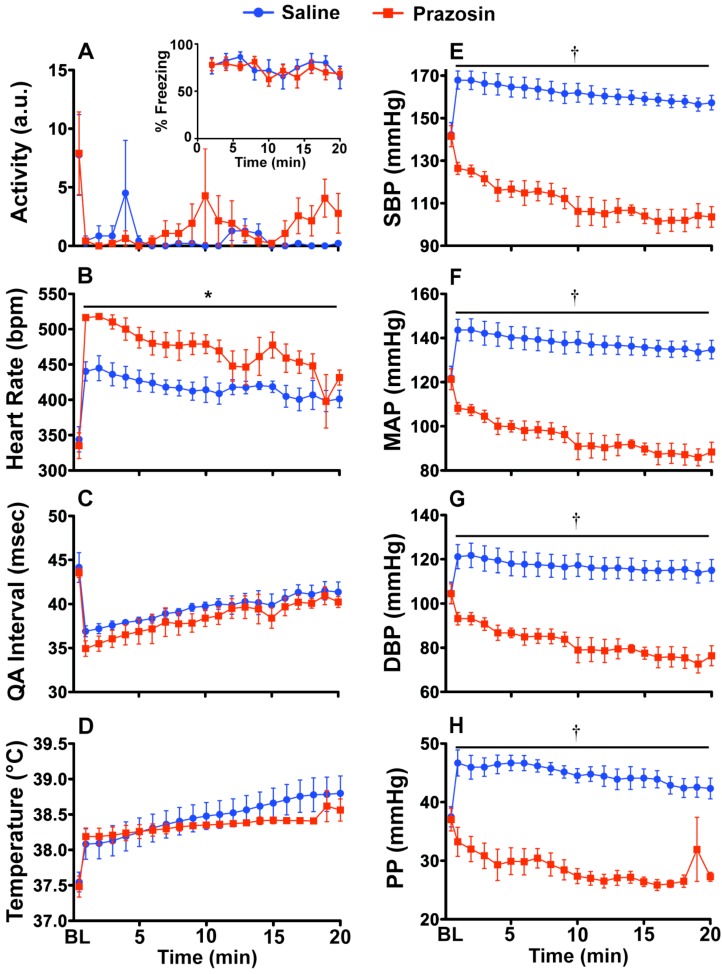
Prazosin (1.0 mg/kg) blocks fear-evoked increases in blood pressure during CFT. *In vivo* telemetry responses during acute CFT. Rats were either injected ip with saline or prazosin 10 min before exposure to the conditioned context. (**A**) Prazosin had no effect on freezing behavior (inset) or locomotor activity; (**B**) Heart rate was increased in prazosin injected rats, likely to compensate for decreased blood pressure; There was no effect of prazosin on the (**C**) QA interval or (**D**) body temperature; (**E–H**) Prazosin prevented the blood pressure increase during exposure to the CFT. Abbreviations are as follows: baseline (BL), arbitrary units (a.u.), systolic blood pressure (SBP), mean arterial pressure (MAP), diastolic blood pressure (DBP), pulse pressure (PP). (*p < 0.05, †.001).

Both behavioral freezing levels (Figure 2A inset) and locomotor activity (Figure 2A) were unaffected by prazosin treatment. Pre-shock baseline levels of freezing ranged between 0–5% (data not shown). Heart rate (Figure 2B) was significantly increased due to prazosin administration (F_(1, 6)_ = 9.118; p = 0.0234). There was no significant effect of prazosin on either the QA interval or on core body temperature (Figure 2C,D). Prazosin completely blocked the stress-induced increase in (Figure 2E–H) SBP (F_(1, 6)_ = 77.016; p = 0.0001), MAP (F_(1, 6)_ = 58.864; p = 0.0003), DBP (F_(1, 6)_ = 34.429; p = 0.0011), and PP (F_(1, 6)_ = 59.848; p = 0.0002). There were significant interactions between drug and time for SBP (F_(19, 114)_ = 77.016; p < 0.0001), MAP (F_(19, 114_) = 58.864; p = 0.0002), and DBP (F_(19, 114)_ = 34.429; p = 0.0008). 

**Figure 3:**
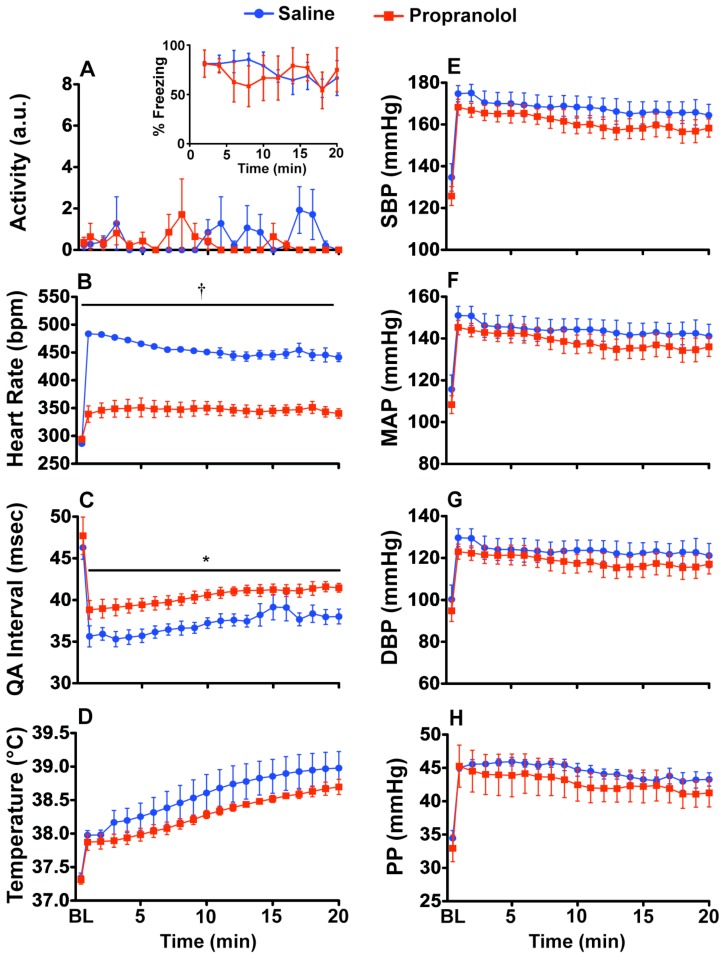
Propranolol (5.0 mg/kg) blocks fear-evoked increases in heart rate. *In vivo* telemetry responses during acute CFT. Rats were either injected ip with saline or propranolol 10 min before exposure to the conditioned context. (**A**) Propranolol had no effect on freezing behavior (inset) or locomotor activity. Pre-shock baseline levels of freezing ranged between 0–5% (data not shown); (**E–H**) Propranolol had no effect on blood pressure during exposure to the CFT; (**B**) Heart rate was decreased in propranolol injected rats; (**C**) The QA interval was significantly increased; (**D**) There was no effect of propranolol on body temperature. Abbreviations are as follows: baseline (BL), arbitrary units (a.u.), systolic blood pressure (SBP), mean arterial pressure (MAP), diastolic blood pressure (DBP), pulse pressure (PP). (* p < 0.05, † p < 0.001).

Both behavioral freezing levels (Figure 3A inset) and locomotor activity (Figure 3A) were unaffected by propranolol treatment. The stress-induced increase in heart rate (Figure 3B) was blocked by propranolol treatment (F_(1, 6)_ = 78.239; p = 0.0001) and there was a time by drug interaction (F_(19,114)_ = 3.128; p < 0.0001). Figure 3C shows a small reduction in the QA interval with propranolol administration (F_(1, 6)_ = 9.317; p = 0.0224). There were no significant differences due to propranolol for core body temperature, SBP, MAP, DBP or PP (Figure 3D-H).

### 2.3. Experiment 3: In vivo Biotelemetry Monitoring during Repeated CFT and 5 Days after Stressor Termination

**Figure 4:**
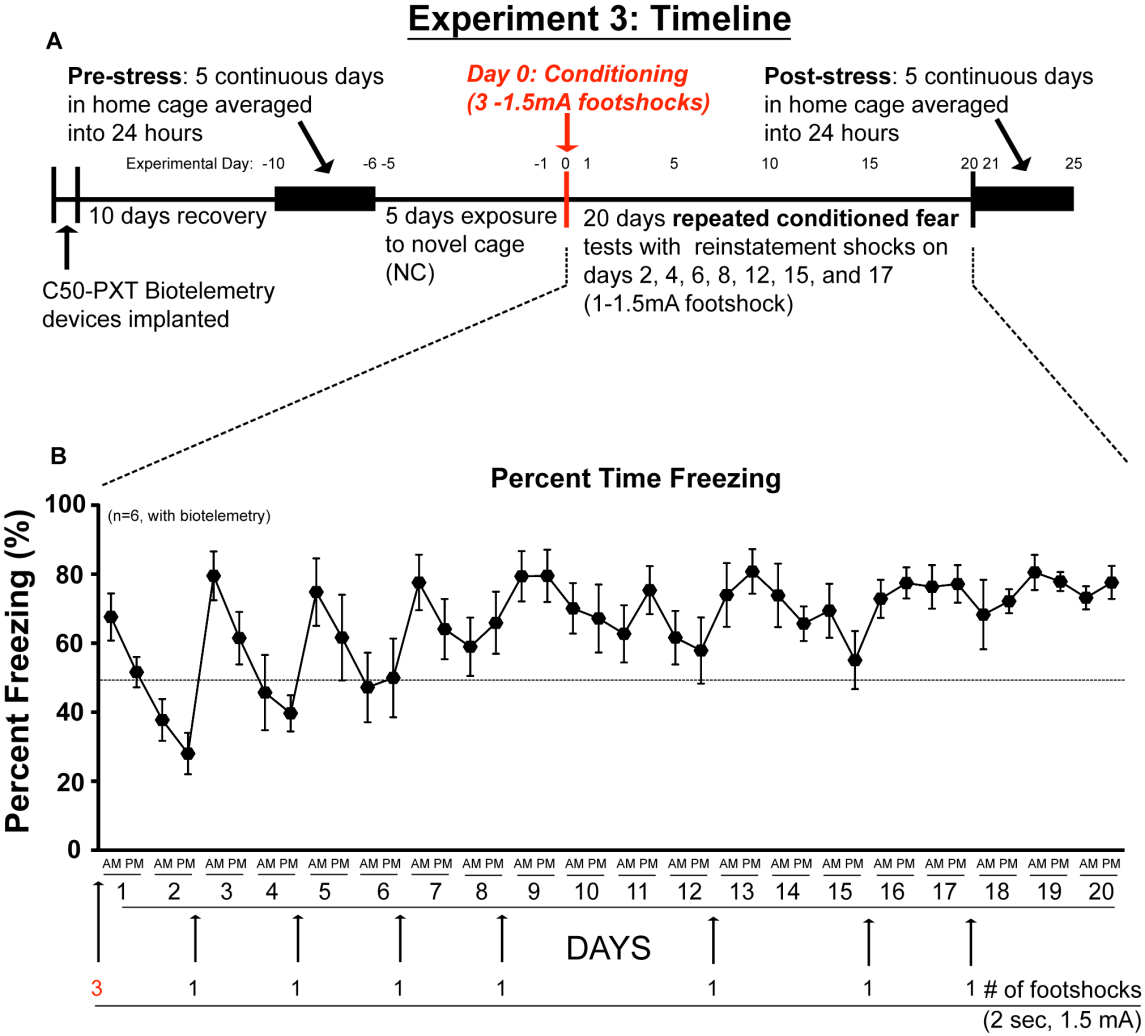
Repeated Exposure to CFT with minimal reinstatement produces consistent fear behavior. Rats were repeatedly exposed to CFT test while simultaneous biotelemetry measurements were taken. Graphs show (**A**) timeline and (**B**) average behavioral freezing response during repeated exposure to the CFT. Pre-shock baseline levels of freezing ranged between 0–5% (data not shown). Abbreviations are as follows: conditioned fear test (CFT).

We again prevented fear extinction with minimal reinstatement footshocks and maintained animals above 50% freezing for most of the duration of repeated CFT (Figure 4).

**Figure 5:**
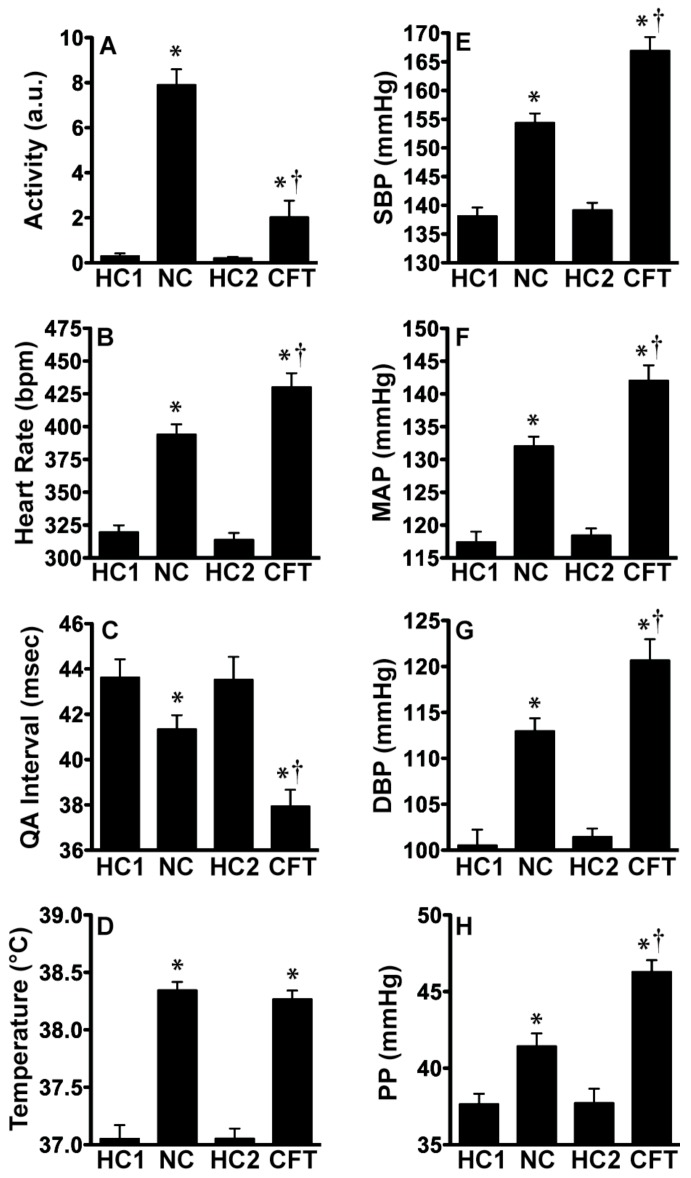
CFT evokes significantly larger cardiovascular responses than novel cage exposure. *In vivo* telemetry measurements demonstrating that (**B**, **C**, **E-H**) cardiovascular responses during CFT were significantly larger than during novel cage exposure despite significantly lower (**A**) activity levels while the (**D**) body temperature responses were similarly increased. There were no significant differences between home cage measurements. Abbreviations are as follows: home cage (HC), HC1 (day −6, Six days before any testing), HC2 (day 0, After five days of novel cage exposure but before any conditioned fear testing), novel cage (NC), conditioned fear test (CFT), arbitrary units (a.u.), systolic blood pressure (SBP), mean arterial pressure (MAP), diastolic blood pressure (DBP), pulse pressure (PP). (* p < 0.05 compared to home cage measurements, † p < 0.05 CFT exposure compared to NC exposure).

Figure 5 reveals that exposure to CFT produced larger cardiovascular responses than exposure to simply a novel cage. There was a significant effect of treatment on locomotor activity (F_(3,15)_ = 61.106; p < 0.0001), heart rate (F_(3,15)_ = 125.663; p < 0.0001), QA interval (F_(3,15)_ = 48.624; p < 0.0001), core body temperature (F_(3,15)_ = 113.022; p < 0.0001), SBP (F_(3,15)_ = 179.864; p < 0.0001), MAP (F_(3,15)_ = 144.369; p < 0.0001), DBP (F_(3,15)_ = 84.05; p < 0.0001), and PP (F_(3,15)_ = 28.186; p < 0.0001). Post hoc analysis using pairwise t-tests with Tukey correction are indicated in Figure 5. Clearly, 5 exposures to the novel cage (NC) evokes an arousal response, howFever, the response is distinguishable from the stress response evoked by CFT. NC produces a greater increase in spontaneous activity than CFT. In addition, NC produces less of an increase in heart rate, blood pressure, and PP than CFT. Thus exposure to the CFT produces greater CV responses in the face of reduced movement.

**Figure 6:**
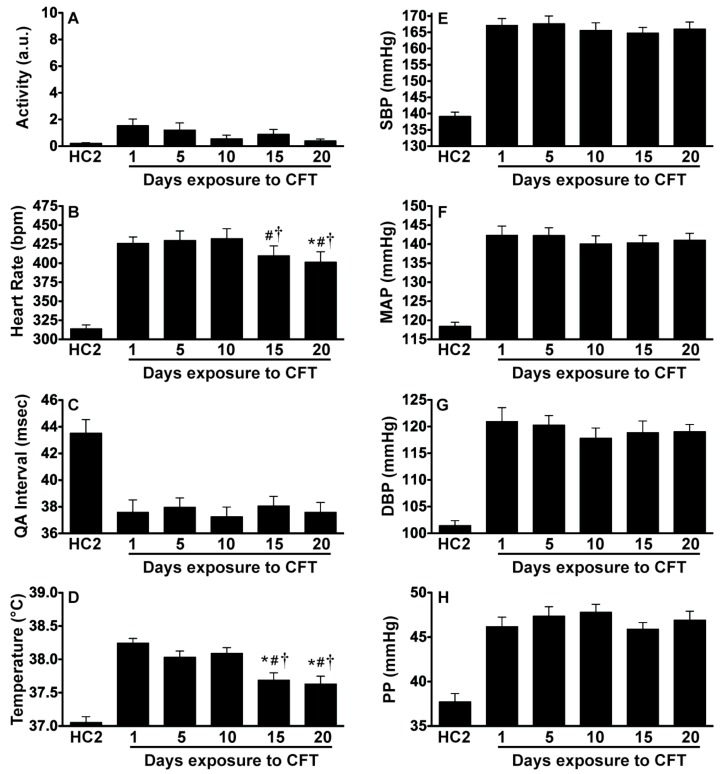
Reduction in fear-evoked increases in heart rate and body temperature with repeated exposure to CFT. *In vivo *telemetry measurements demonstrating that (**A**) locomotor activity was consistently low and not significantly different with repeated exposures to CFT; (**B**) Heart rate was consistently elevated but this response was reduced with repeated exposures to CFT; (**C**) The QA interval was consistently reduced with no alterations due to repeated exposure; (**D**) Body temperature was consistently increased but this response was reduced with repeated exposures to CFT; (**E–H**) Blood pressure was consistently elevated and this response did not habituate with repeated exposures to CFT. Abbreviations are as follows: home cage (HC), conditioned fear test (CFT), arbitrary units (a.u.), systolic blood pressure (SBP), mean arterial pressure (MAP), diastolic blood pressure (DBP), pulse pressure (PP). (* p < 0.05 compared to Day 1, # p < 0.05 compared to Day 5, † p < 0.05 compared to Day 10).

Locomotor activity, QA interval, SBP, MAP, DBP, and PP were significantly elevated above both home cage measurements and novel cage exposure (ps < 0.0001). Importantly, these responses were repeatable and sustainable across multiple sessions. There were no significant changes of these responses with repeated exposure to CFT. In contrast, heart rate (F_(4, 20)_ = 8.46; p = 0.0004) and core body temperature (F_(4,20)_ = 13.15; p < 0.0001) did decline with repeated exposures to CFT. Post hoc analysis using pairwise t-tests with Tukey correction are denoted in Figure 6.

**Figure 7:**
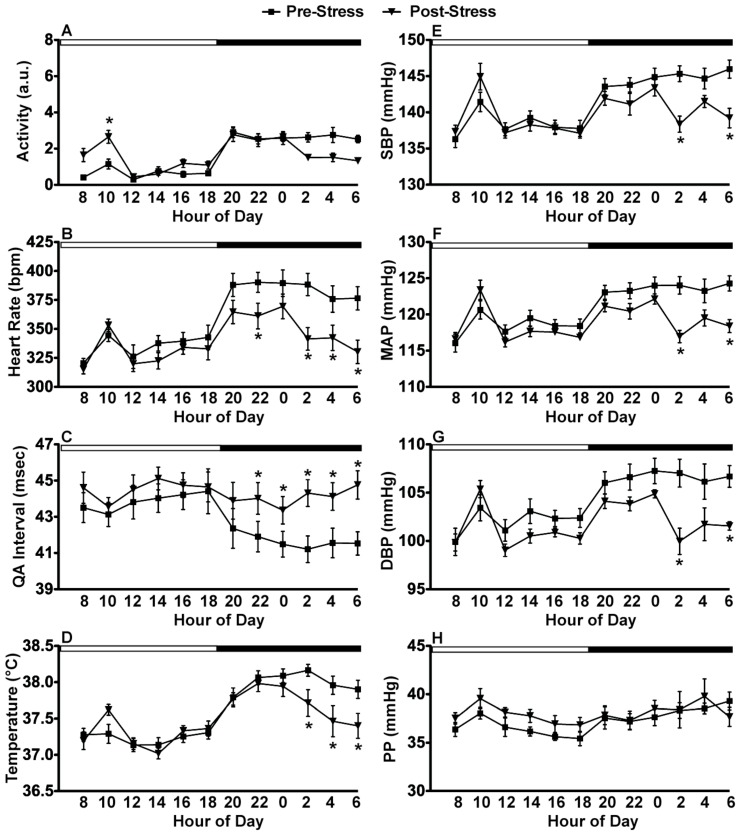
Diurnal disruptions produced by repeated exposure to CFT. *In vivo* telemetry measurements in the home cage graphed in 2-hr blocks demonstrate the consequences of 20 days (40 exposures) of CFT. (**A**) Activity was significantly increased during the day but there was only a trend towards a decrease during the night; (**B**) Heart rate; (**D**) body temperature; and (**E–H**) blood pressure were also significantly reduced during the latter part of the night; (**C**) The QA interval was significantly increased during the night post-stress; (**H**) There was no effect on pulse pressure. Abbreviations are as follows: arbitrary units (a.u.), systolic blood pressure (SBP), mean arterial pressure (MAP), diastolic blood pressure (DBP), pulse pressure (PP), conditioned fear test (CFT). (* p < 0.05 compared to pre-stress values).

Exposure to CFT produced changes in the 24-hour diurnal rhythm responses measured pre- and post-stress in undisturbed rats in their home cages. Time of day analysis showed there were significant effects post-stress on locomotor activity (F_(23, 115)_ = 14.34; p < 0.0001), heart rate (F_(23, 115)_ = 21.05; p < 0.0001), QA interval (F_(23, 115)_ = 16.05; p < 0.0001), core body temperature (F_(23, 115)_ = 22.28; p < 0.0001), SBP (F_(23, 115)_ = 11.46; p < 0.0001), MAP (F_(23, 115)_ = 10.88; p < 0.0001), DBP (F_(23, 115)_ = 8.06; p < 0.0001) and PP (F_(23, 115)_ = 2.34; p = 0.0017). Post hoc analysis using pairwise t-tests with Tukey correction are denoted in Figure 7.

**Figure 8:**
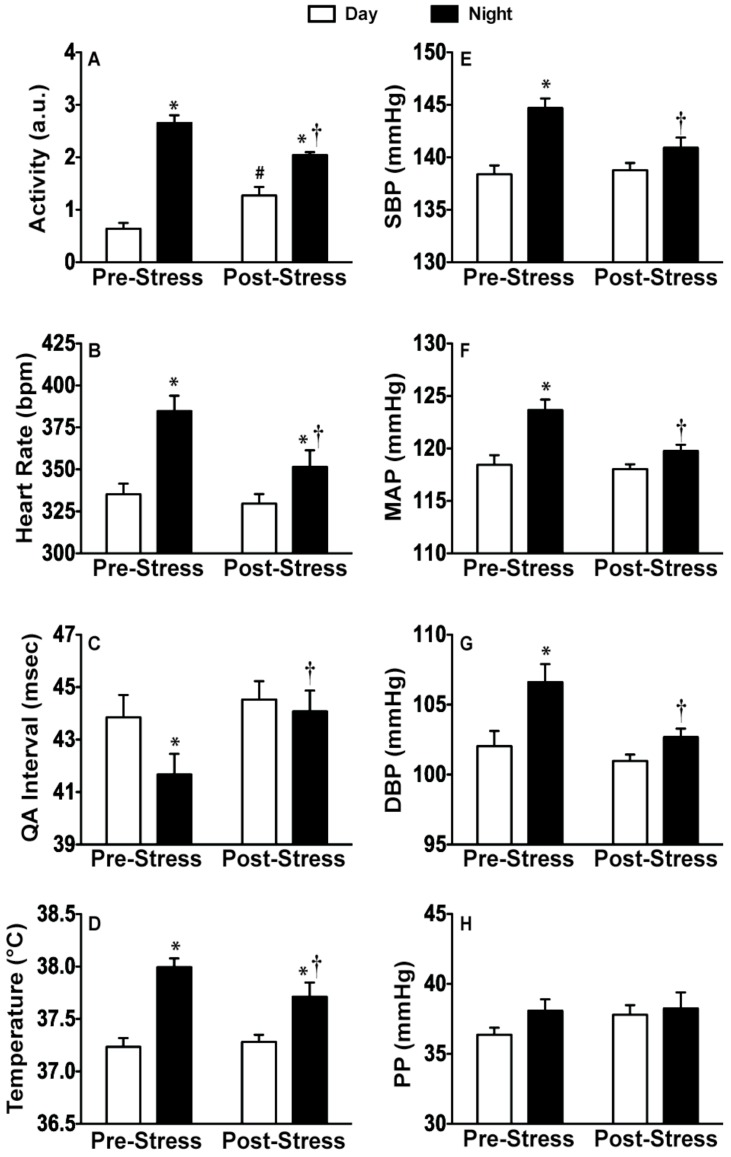
Flattening of diurnal rhythm. Day/night averages both pre-stress and post-stress demonstrating significant reductions in diurnal variation in measured variables post-stress. (**A**) Activity was significantly increased in the day and reduced in the night post-stress; (**B**) Heart rate; (**D**) body temperature; and (**E–G**) blood pressure were all significantly reduced during the night post-stress; (**C**) The QA interval was significantly increased during the night post-stress; (**H**) There was no effect on pulse pressure. Abbreviations are as follows: arbitrary units (a.u.), systolic blood pressure (SBP), mean arterial pressure (MAP), diastolic blood pressure (DBP), and pulse pressure (PP). (* p < 0.05 compared to respective day values, † p < 0.05 compared to pre-stress night, # p < 0.05 compared to pre-stress day value).

If we collapse the results across 12-h blocks, the day/night analysis revealed that the diurnal variation of locomotor activity (F_(3, 15)_ = 83.12; p < 0.0001), heart rate (F_(3, 15)_ = 41.13; p < 0.0001), QA interval (F_(3, 15)_ = 41.48; p < 0.0001), core body temperature (F_(3, 15)_ = 82.5; p < 0.0001), SBP (F_(3, 15)_ = 18.95; p < 0.0001), MAP (F_(3, 15)_ = 16.52; p < 0.0001), and DBP (F_(3, 15)_ = 10.01; p < 0.0001), but not PP, was flattened post-stress. Post hoc analysis using pairwise t-tests with Tukey correction are indicated in Figure 8.

### 2.4. Discussion of Results

Our evidence is consistent with the conclusion that CFT produced vascular dominated MAP by altering total peripheral vascular resistance (TPR). Vascular dominated blood pressure can occur when the blood pressure response is the same but the heart rate response is reduced suggesting that the contribution of changes in peripheral vascular resistance is a dominant contributor to MAP levels. We base this on several lines of evidence. First, it is clear that CFT initially evoked strong tachycardia, but that this response was reduced by day 20. Interestingly, despite a reduced HR on day 20, the MAP response during CFT for day 1 *vs*. day 20 was equal (Figure 7). This effect could be due to increased stroke volume (SV), increased TPR, and/or baroreceptor resetting. Stroke volume is one component of cardiac output (CO) and HR is the other such that CO = HR × SV. In order to maintain an increase in MAP with a reduced HR, either SV had to increase, TPR needed to increase, or some combination of both needed to occur (MAP = (HR × SV) × TPR). Increases in SV can result from increases in cardiac contractility [56]. The QA interval is an estimate of cardiac contractility [57,58] and these are inversely related such that a decreased QA interval suggests increased cardiac contractility. Given there was no change in the cardiac contractility responses after repeated CFT (Figure 6C), it is likely SV was not changed by the stress experience. Although SV was not measured directly and thus we cannot rule out its contribution to this effect, it is reasonable to suggest that increased TPR played a larger role in altering the CV dynamics during repeated CFT. This type of vascular dominated MAP support can be due to increased sympathetic tone or vascular stiffening leading to changes in TPR, and has been reported to be a preclinical marker of reduced CV health [16,17,19,59]. 

In addition to changes in TPR, reduced HR and sustained MAP could also involve changes in baroreflex circuitry. In response to increased blood pressure, the baroreflex circuit causes withdrawal of sympathetic drive and a simultaneous increase in parasympathetic drive to the heart, effectively lowering heart rate [60,61]. Conti *et al*. reported that repeatedly restrained rats have similar changes in blood pressure responses to phenylephrine (an α_1_ receptor agonist) and larger reflexive reductions in heart rate [62]. This suggests that repeated stress may increase baroreceptor sensitivity. Increased baroreceptor sensitivity could explain why heart rate was reduced on day 20 compared to day 1. Thus, changes in baroreceptor reflex circuitry may have, in part, played a role in reducing the heart rate response on day 20 of repeated CFT.

Exposure to CFT increased core body temperature or evoked stress-induced hyperthermia (SIH). SIH was demonstrable during CFT on all days tested, which is consistent with previous reports [39,43,63]. Interestingly, the SIH response decreased with repeated CFT exposures and was reliably reduced after 15 days of CFT (Figure 6D). According to blood flow measurements, the heart muscle itself accounts for approximately 20% of thermogenesis in the rat [63,64]. Thus, the decrease in HR that occurs after CFT may contribute to the observed reduction in SIH. 

Repeated exposure to CFT also produced profound changes in diurnal rhythms. When diurnal rhythms are disrupted, disorders of diverse physiological processes can occur [65]. In addition, diurnal disruption (*i.e.*, amplitude reduction or phase shifting) is frequently co-morbid with many stress-sensitive diseases including cancer, obesity and affective disorders [65]. We demonstrated significant decreases in activity, blood pressure, heart rate, cardiac contractility, and body temperature during the latter part of the active cycle suggesting that repeated CFT during the inactive cycle disrupted normal diurnal variations (Figure 7 and Figure 8). Diurnal disruptions evoked by stressor exposure may contribute to the regularity of cardiac events, like myocardial infarction and cardiac death, at specific times of the day [66,67,68]. Our data suggests that repeated CFT produces flattened diurnal rhythms, which can be indicative of increased disease susceptibility. One strength of this experimental approach using continuous biotelemetric recording is that the data demonstrate no detectable phase shift in the rhythms of animals exposed to repeated CFT, adding to one’s confidence that CFT truly produced diurnal rhythms flattening. Thus, the changes produced by repeated CFT provides an experimental approach for investigating the role of stress-induced diurnal disruption on the CV system. 

## 3. Experimental Section

### 3.1. Animals

Adult male Sprague-Dawley rats (n = 36, Harlan Laboratories) weighing 200 g– 230 g were used in our experiments and housed in a pathogen free barrier facility with controlled temperature (22 °C) and humidity. The animals were maintained on a 12:12 hour light/dark cycle (lights on 0700–1900 hours). Animals acclimated to housing conditions for one week before any manipulations occurred. All rats were sedentary and housed in Nalgene Plexiglas cages (45 × 25.2 × 14.7 cm). Rats had *ad libitum* access to food and water and were weighed daily. All experimental procedures were performed during the inactive cycle and animals were handled during the 1-week acclimation period. Animal discomfort was minimized during all procedures. Experimental protocols for these studies were approved by the University of Colorado Animal Care and Use Committee.

### 3.2. Biotelemetry Surgeries

The C50-PXT biotelemetry transmitters (Data Sciences International, St. Paul Minnesota) were implanted into animals as previously described [44,69]. Briefly, animals were fully anesthetized and unresponsive following ketamine (i.p. 75.0 mg/kg), and medetomidine (i.p. 0.5 mg/kg). Animals were shaved and prepped for surgery. Body temperature was maintained and monitored on a heating pad throughout surgery. A midline incision was made approximately 5.0 cm in length on the ventral abdominal wall. Intestines were gently moved and the abdominal aorta isolated. The abdominal aorta was occluded rostral to the catheter entry site. Once occluded, the blood pressure catheter was inserted into the abdominal aorta and secured in place with a cellulose patch (Data Sciences International, St. Paul Minnesota) and glue (3M Vetbond Adhesive). The intestines were gently floated back into place with sterile saline and the C50-PXT transmitter was sutured into the ventral abdominal wall. Finally, the ECG leads were sutured into place to measure cardiac electrical activity. Animals were allowed to recover for 10 days before recording began. 

### 3.3. Data Acquisition and Analysis

The C50-PXT transmitter allows *in vivo *real time measurement of locomotor activity (LA), heart rate (HR), QA Interval (QAI), core body temperature (CBT), systolic blood pressure (SBP), mean arterial pressure (MAP), diastolic blood pressure (DBP) and pulse pressure (PP). The QAI is a measure of the time (milliseconds) between the Q-wave (Q) of the QRS complex and the onset of the aortic pulse (A) and can be used as an estimation of cardiac contractility. There is an inverse relationship between QAI and cardiac contractility such that a decrease in QAI is suggestive of increased cardiac contractility [57,58]. Biotelemetry recordings were acquired/analyzed using Dataquest ART 4.3 Gold Acquisition/Analysis Software (Data Sciences International, St. Paul, MN). Blood pressure, heart rate, and locomotor activity were recorded at 500 Hz.

### 3.4. Contextual Conditioned Fear Test (CFT)

During acute CFT (Experiment 2) rats were exposed to a context or conditioned stimulus followed by exposure to footshock or unconditioned stimulus. Later when rats were re-exposed to this same context or conditioned stimulus they displayed fear behavior in the form of freezing (measured by an observer) in the absence of the unconditioned stimulus [37,38,39]. Freezing behavior was defined as all four paws on the shuttle box grid floor and an absence of movement except for that necessary for respiration [37,38]. Rats were placed into the shuttle box (46 × 20.7 × 20 cm) on *day 0 *for 5 min to acquire a memory of the context environment, as previously described [70], after which one 2-sec 1.5 mA footshock was administered at 5, 6, and 7 min and occurred at 1000 hrs. Subsequently rats were returned to the home cage environment. Twenty-four hours later, *day 1*, behavioral freezing was measured during a 20-min re-exposure to the shuttle box in the absence of footshock. 

During repeated CFT (Experiment 3) rats were first conditioned as stated above and were exposed to the conditioned context during the daylight portion of their diurnal cycle on a random variable time scale once for 20-min in the morning (AM) and once for 20-min in the afternoon (PM). No significant differences were found in the responses between AM and PM tests; therefore, the biotelemetry data were averaged for clarity. Rats were exposed to CFT 2 times per day for the next 20 days (Figure 1 and Figure 4). Extinction of fear behavior (freezing) occurs if rats are repeatedly exposed to context in the absence of footshock [71,72,73]. To prevent extinction, minimal numbers of reinstatement footshocks were used to re-pair the shuttlebox environment and footshock [70,71,72]. To maintain high levels of freezing, we administered a single reinstatement footshock (2 sec–1.5 mA) when group freezing in the shuttle box was estimated to drop below 50% during the PM test. The reinstatement footshock was administered at the end of the PM test. We (data not shown) and others [74] have found that rats demonstrating freezing above 50% to conditioned context have a significant increase in blood catecholamines. A total of 5 reinstatement footshocks (Figure 1B) were used in Experiment 1 and 7 reinstatement footshocks were used in Experiment 3 (Figure 4B). The total amount of time rats actually received footshock for Experiment 1 was 16-sec across 20 days and for Experiment 3 was 20-sec across 20 days.

### 3.5. Experiment 1: Repeated Exposure to CFT and Tissue Collection

Fourteen rats were exposed to repeated CFT and sacrificed for tissues (without biotelemetry transmitters). As described by the classic work of Hans Selye, physiological changes that are indicative of chronic activation of the stress response include increased blood levels of corticosterone, decreased body weight, thymic involution, and adrenal hypertrophy [75]. We measured these variables in sacrificed animals the morning following the last CFT. A total of 6 rats were repeatedly exposed to CFT and 8 rats were used as home cage controls.

Animals were rapidly decapitated for blood, thymus and adrenal collection. Blood was collected in EDTA tubes and immediately spun at 3000 g in a refrigerated (4 °C) centrifuge for 15 min. Plasma was removed and frozen in a −80 °C freezer (Legaci Refrigeration System-Kendro Laboratory Products, Asheville NC USA) for later analysis. The thymus and adrenals were immediately microdissected from each animal to remove excess tissue and weighed.

Corticosterone levels in the plasma were measured using a commercially available EIA Kit (Enzo Life Sciences) as previously described [76]. Briefly, plasma (10.0µL) was diluted 1:50 with assay buffer to a final volume of 100.0 µL for use in the EIA Kit. The diluted sample was then processed according to the EIA kit directions. Plasma or corticosterone standard was incubated in a 96-well plate with standard diluent. After 2-h incubation on a plate shaker (500 rpm), the plate was washed to clear unbound reagent and substrate was added. After 1-h incubation the reaction was stopped and read with a microplate reader (Molecular Devices, E_max_ precision microplate reader) at 405 nm. Concentrations were compared against the standard curve and expressed as micrograms per deciliter (µg/dL) of serum.

### 3.6. Experiment 2: Verification of SNS Involvement during CFT

Sixteen rats were used for the drug studies during acute CFT. Fear-evoked increases in heart rate and blood pressure are due to activation of the SNS [40,41,42,77]. To verify that our fear-evoked responses are also mediated, in part, by activation of the SNS, we tested blockade of α_1_-adrenergic receptors (in blood vessels) or β-adrenergic receptors (in cardiac tissue). Rats were implanted with biotelemetry transmitters and allowed one week to recover before experiments began. We injected the α_1_-adrenergic receptor antagonist Prazosin (i.p. 1.0 mg/kg) into animals (n = 4 animals/group) 10 min before CFT (Figure 2). We then injected the β-adrenergic receptor antagonist Propranolol (i.p. 5.0 mg/kg) into animals (n = 4 animals/group) 10 min before CFT (Figure 3). Drugs were dissolved in 0.9% saline (Hospira, Inc., Lake Forest, IL, USA) and saline injections were used as the control for both studies. For comparison, baseline measurements were collected from 30 min before entry into the animal room before drug administration.

### 3.7. Experiment 3: Biotelemetry Monitoring during Repeated CFT

Six rats were implanted with biotelemetry transmitters and allowed 1-week to recover before baseline recordings began. Figure 4 shows the experimental timeline for repeated exposure to CFT. Baseline diurnal measurements were recorded and averaged, from *days *−*10* through −*6,* into one 24-h interval to serve as a pre-stress baseline. LA, HR, QAI, blood pressure and CBT were measured continuously in the home cage for these five days prior to any experimental manipulation in order to acquire baseline diurnal variation in our animals. Two separate baseline measurements were obtained in the home cage (HC) from an average of 2-hrs (700–900 hrs). The first measurement was on day −6 (HC1) before novel cage exposure and the second measurement was on day 0 (HC2) after novel cage exposure but before exposure to CFT (Figure 4). On days −5 through −1 animals were moved to a novel cage (26.5 × 16.5 × 11.5 cm) for 20 min once per day which was located outside of the home cage vivarium. During repeated exposure to the novel cage (NC) environment LA, HR, QAI, blood pressure, and CBT were averaged and used to calculate representative physiological responses during exposure to a benign environment and served to accurately reflect fluctuations in blood pressure and HR caused by repeated handling and transport of the animals into another room. First, we measured time-matched baseline physiology from rats in their home cages (HC1, HC2). HC1 was calculated from Day −6, *i.e*., after surgical recovery but before any additional behavioral testing. HC2 was calculated from Day 0, *i.e*., after 5 days of NC exposure but before any CFT. Then we assessed the average effect of repeated exposures to a NC (Days −5 to −1) and the average effect of repeated exposures to CFT (Days 1 to 5) on biotelemetric physiology. Subsequently, we examined whether responses were altered with 20 days repeated exposure to CFT. Finally, after 20 days of repeated CFT, 5 days of continuous data (days 21–25) were collected in the home cage and averaged into one 24-hr interval (post-stress) to compare with the pre-stress 24-hr baseline.

### 3.8. Statistical Analysis

For experiment 1, ANOVA was used to examine the differences between home cage control and repeated CFT. For experiment 2, repeated measures ANOVA was used comparing either drug or saline. For experiment 3, repeated measures ANOVA was run on individual treatment of the animals defined as HC1 then NC exposure, then HC2, and lastly CFT. Repeated measures ANOVA was performed on repeated exposures to CFT (Days 1, 5, 10, 15, and 20). Finally, repeated measures ANOVA was run for time of day and day/night analysis. When repeated measures ANOVA yielded significant results, post hoc analysis using pairwise t-tests with Tukey correction were performed. 

## 4. Conclusions

The current experiments demonstrate that 20 days of CFT produces changes that are classically associated with chronic or repeated stressor exposure, *i.e*., decreased body weight, decreased thymus weight, increased adrenal weight, elevated basal CORT, and flattened diurnal rhythms. The blood pressure and heart rate increases evoked during CFT are greater than responses produced by exposure to a novel environment or handling. These responses are driven by the SNS with α_1_-adrenergic receptors (in blood vessels) mediating blood pressure increases, and β-adrenergic receptors (most likely in cardiac tissue) mediating heart rate increases. The increases in cardiovascular responses and CBT during CFT are likely due to the emotional fear response, and are not a consequence of changes in activity as they occur in the absence of increased levels of activity during the test. Consistent with previous work [78], the cardiovascular and body temperature responses did not extinguish after repeated CFT with reinstatement. Thus, repeated exposure to CFT with reinstatement and *in vivo* biotelemetry monitoring is an experimental approach that triggers repeatable fear responses and fear-evoked SNS mediated cardiovascular responses without marked extinction of the response. Finally, repeated exposure to CFT is sufficient to produce vascular dominated MAP (a pre-clinical marker of CV dysfunction) and flattened diurnal rhythms (a common feature of many stress-related mood disorders) and provides an optimal tool to unravel the relationships between the physiological and potential health consequences of repeated/chronic emotional stress.

We feel our work makes an important novel contribution for several reasons. First, there is a rich literature investigating conditioned fear; however, fewer examples measuring real-time *in vivo* physiological measures, including 24-hr day/night measures. In fact, most studies using conditioned fear present their physiological data as a change score, which complicates interpretation of the data. No one has ever simultaneously examined both the behavioral and the physiological consequences of repeated exposures to the conditioned fear paradigm and as such, both our behavioral data (*i.e*., freezing) and physiological results provide for the first time a clear picture demonstrating exactly how rats respond in real-time both behaviorally and physiologically to repeated CFT.

Second, while other paradigms have examined similar responses to repeated stressors, using conditioned fear takes advantage of the literature in that a very specific neurocircuit is known to underlie both the behavioral and physiological consequences of exposure to an environment a rat is conditioned to fear (*i.e*., basolateral amygdala, central amygdala, caudal vlPAG, dorsomedial hypothalamus, raphé pallidus, and rostral ventrolateral medulla). More importantly based on known literature, future experiments can examine how components of this neurocircuit are altered with repeated exposures to CFT. This will further our understanding of how neural alterations may produce negative physiological consequences that can underlie the development of disease.

Finally, using repeated CFT in conjunction with biotelemetry allows examination of diurnal rhythms as well as circadian rhythms (if constant dark conditions are studied). In fact, subsequent experiments using repeated CFT (in prep) have been performed examining this very question. Not only can fear conditioning negatively affect rhythms, but subsequent stressor exposure significantly alters both stress responses and diurnal rhythms in animals with prior repeated CFT. 

## References

[1] Silverthorn D.U. (2007). Human Physiology: An Integrated Approach.

[2] Forsman L., Lindblad L.E. (1983). Effect of mental stress on baroreceptor-mediated changes in blood pressure and heart rate and on plasma catecholamines and subjective responses in healthy men and women. Psychosom. Med..

[3] Hamer M., Gibson E.L., Vuononvirta R., Williams E., Steptoe A. (2006). Inflammatory and hemostatic responses to repeated mental stress: Individual stability and habituation over time. Brain, Behav. Immunity.

[4] Hjemdahl P., Fagius J., Freyschuss U., Wallin B.G., Daleskog M., Bohlin G., Perski A. (1989). Muscle sympathetic activity and norepinephrine release during mental challenge in humans. Am. J. Physiol..

[5] Kubzansky L.D., Adler G.K. (2010). Aldosterone: A forgotten mediator of the relationship between psychological stress and heart disease. Neurosci. Biobehav. Rev..

[6] Cohen S., Janicki-Deverts D., Miller G.E. (2007). Psychological stress and disease. JAMA: J. Am. Med. Assoc..

[7] Dimsdale J.E. (2008). Psychological stress and cardiovascular disease. J. Am. Coll. Cardiol..

[8] Rozanski A., Blumenthal J.A., Davidson K.W., Saab P.G., Kubzansky L. (2005). The epidemiology, pathophysiology, and management of psychosocial risk factors in cardiac practice: The emerging field of behavioral cardiology. J. Am. Coll.Cardiol..

[9] Grippo A.J., Moffitt J.A., Johnson A.K. (2008). Evaluation of baroreceptor reflex function in the chronic mild stress rodent model of depression. Psychosom. Med..

[10] Iso H., Date C., Yamamoto A., Toyoshima H., Tanabe N., Kikuchi S., Kondo T., Watanabe Y., Wada Y., Ishibashi T. (2002). Perceived mental stress and mortality from cardiovascular disease among japanese men and women: The japan collaborative cohort study for evaluation of cancer risk sponsored by monbusho (jacc study). Circulation.

[11] Rosengren A., Tibblin G., Wilhelmsen L. (1991). Self-perceived psychological stress and incidence of coronary artery disease in middle-aged men. Am. J. Cardiol..

[12] Smith T.W., Ruiz J.M. (2002). Psychosocial influences on the development and course of coronary heart disease: Current status and implications for research and practice. J. Consult. Clin. Psychol..

[13] Grippo A.J., Johnson A.K. (2009). Stress, depression and cardiovascular dysregulation: A review of neurobiological mechanisms and the integration of research from preclinical disease models. Stress.

[14] Baumert M., Schlaich M.P., Nalivaiko E., Lambert E., Sari C.I., Kaye D.M., Elser M.D., Sanders P., Lambert G. (2011). Relation between qt interval variability and cardiac sympathetic activity in hypertension. Am. J. Physiol. Heart Circ. Physiol..

[15] Nalivaiko E. (2011). Animal models of psychogenic cardiovascular disorders: What we can learn from them and what we cannot. Clin. Exp. Pharmacol. Physiol..

[16] Branch C.A., Knuepfer M.M. (1994). Causes of differential cardiovascular sensitivity to cocaine. Ii: Sympathetic, metabolic and cardiac effects. J. Pharmacol. Exp. Ther..

[17] Knuepfer M.M., Purcell R.M., Gan Q., Le K.M. (2001). Hemodynamic response patterns to acute behavioral stressors resemble those to cocaine. Am. J. Physiol. Regul. Integr. Comp. Physiol..

[18] Light K.C., Dolan C.A., Davis M.R., Sherwood A. (1992). Cardiovascular responses to an active coping challenge as predictors of blood pressure patterns 10 to 15 years later. Psychosom. Med..

[19] Muller J.R., Le K.M., Haines W.R., Gan Q., Knuepfer M.M. (2001). Hemodynamic response pattern predicts susceptibility to stress-induced elevation in arterial pressure in the rat. Am. J. Physiol. Regul. Integr. Comp. Physiol..

[20] Chrousos G.P. (2000). Stress, chronic inflammation, and emotional and physical well-being: Concurrent effects and chronic sequelae. J. Allergy Clin. Immunol..

[21] Pertsov S.S., Koplik E.V., Stepanyuk V.L., Simbirtsev A.S. (2009). Blood cytokines in rats with various behavioral characteristics during emotional stress and treatment with interleukin-1beta. Bull. Exp. Biol. Med..

[22] Picardi A., Mazzotti E., Gaetano P., Cattaruzza M.S., Baliva G., Melchi C.F., Biondi M., Pasquini P. (2005). Stress, social support, emotional regulation, and exacerbation of diffuse plaque psoriasis. Psychosomatics.

[23] Shao F., Lin W., Wang W., Washington W.C., Zheng L. (2003). The effect of emotional stress on the primary humoral immunity of rats. J. Psychopharmacol..

[24] Amiragova M.G. (1985). Neurophysiological analysis of the development of endocrine and hypertensive reactions in prolonged emotional stress. Brain Res..

[25] Shoji H., Mizoguchi K. (2010). Acute and repeated stress differentially regulates behavioral, endocrine, neural parameters relevant to emotional and stress response in young and aged rats. Behav. Brain Res..

[26] Batalha V.L., Pego J.M., Fontinha B.M., Costenla A.R., Valadas J.S., Baqi Y., Radjainia H., Muller C.E., Sebastiao A.M., Lopes L.V. (2012). Adenosine a(2a) receptor blockade reverts hippocampal stress-induced deficits and restores corticosterone circadian oscillation. Mol. Psychiatry.

[27] Christiansen S., Bouzinova E.V., Palme R., Wiborg O. (2012). Circadian activity of the hypothalamic-pituitary-adrenal axis is differentially affected in the rat chronic mild stress model of depression. Stress.

[28] Koresh O., Kozlovsky N., Kaplan Z., Zohar J., Matar M.A., Cohen H. (2012). The long-term abnormalities in circadian expression of period 1 and period 2 genes in response to stress is normalized by agomelatine administered immediately after exposure. Eur. Neuropsychopharmacol..

[29] Richards R.S., Nwose E.U., Bwititi P. (2011). Biochemical basis of circadian rhythms and diseases: With emphasis on post-traumatic stress disorder. Med. Hypotheses.

[30] Arble D.M., Ramsey K.M., Bass J., Turek F.W. (2010). Circadian disruption and metabolic disease: Findings from animal models. Best Pract. Res. Clin. Endocrinol. Metab..

[31] Albrecht U. (2012). Circadian rhythms and sleep—The metabolic connection. Pflugers Arch..

[32] Takeda N., Maemura K. (2011). Circadian clock and cardiovascular disease. J. Cardiol..

[33] Portaluppi F., Tiseo R., Smolensky M.H., Hermida R.C., Ayala D.E., Fabbian F. (2012). Circadian rhythms and cardiovascular health. Sleep Med. Rev..

[34] McClung C.A. (2007). Clock genes and bipolar disorder: Implications for therapy. Pharmacogenomics.

[35] McClung C.A. (2007). Circadian genes, rhythms and the biology of mood disorders. Pharmacol. Ther..

[36] Bunney J.N., Potkin S.G. (2008). Circadian abnormalities, molecular clock genes and chronobiological treatments in depression. Br. Med. Bull..

[37] Blanchard D.C., Blanchard R.J. (1972). Innate and conditioned reactions to threat in rats with amygdaloid lesions. J. Comp. Physiol. Psychol..

[38] Fanselow M.S., Gale G.D. (2003). The amygdala, fear, and memory. Ann. N. Y. Acad. Sci..

[39] Godsil B.P., Quinn J.J., Fanselow M.S. (2000). Body temperature as a conditional response measure for pavlovian fear conditioning. Learn. Mem..

[40] Carrive P. (2000). Conditioned fear to environmental context: Cardiovascular and behavioral components in the rat. Brain Res..

[41] Carrive P. (2002). Cardiovascular and behavioural components of conditioned fear to context after ganglionic and alpha-adrenergic blockade. Auton. Neurosci..

[42] Choi E.A., Leman S., Vianna D.M., Waite P.M., Carrive P. (2005). Expression of cardiovascular and behavioural components of conditioned fear to context in t4 spinally transected rats. Auton. Neurosci..

[43] Vianna D.M., Carrive P. (2005). Changes in cutaneous and body temperature during and after conditioned fear to context in the rat. Eur. J. Neurosci..

[44] Carrive P. (2006). Dual activation of cardiac sympathetic and parasympathetic components during conditioned fear to context in the rat. Clin. Exp. Pharmacol. Physiol..

[45] Dias-Ferreira E., Sousa J.C., Melo I., Morgado P., Mesquita A.R., Cerqueira J.J., Costa R.M., Sousa N. (2009). Chronic stress causes frontostriatal reorganization and affects decision-making. Science.

[46] Vandekerckhove M., Cluydts R. (2010). The emotional brain and sleep: An intimate relationship. Sleep Med. Rev..

[47] Nishino S. (2011). Hypothalamus, hypocretins/orexin, and vigilance control. Handb. Clin. Neurol..

[48] Morimoto K., Tan N., Nishiyasu T., Sone R., Murakami N. (2000). Spontaneous wheel running attenuates cardiovascular responses to stress in rats. Pflugers Arch..

[49] Murphy H.M., Wideman C.H., Aquila L.A., Nadzam G.R. (2002). Telemetry provides new insights into entrainment of activity wheel circadian rhythms and the role of body temperature in the development of ulcers in the activity-stress paradigm. Integr. Physiol. Behav. Sci..

[50] Soszynski D., Kozak W., Conn C.A., Rudolph K., Kluger M.J. (1996). Beta-adrenoceptor antagonists suppress elevation in body temperature and increase in plasma il-6 in rats exposed to open field. Neuroendocrinology.

[51] Leman S., Dielenberg R.A., Carrive P. (2003). Effect of dorsal periaqueductal gray lesion on cardiovascular and behavioural responses to contextual conditioned fear in rats. Behav. Brain Res..

[52] Glavin G.B., Pare W.P., Sandbak T., Bakke H.K., Murison R. (1994). Restraint stress in biomedical research: An update. Neurosci. Biobehav. Rev..

[53] Simpkiss J.L., Devine D.P. (2003). Responses of the hpa axis after chronic variable stress: Effects of novel and familiar stressors. Neuroendocrinol. Lett..

[54] Babygirija R., Bulbul M., Cerjak D., Ludwig K., Takahashi T. (2011). Sustained acceleration of colonic transit following chronic homotypic stress in oxytocin knockout mice. Neurosci. Lett..

[55] Girotti M., Pace T.W., Gaylord R.I., Rubin B.A., Herman J.P., Spencer R.L. (2006). Habituation to repeated restraint stress is associated with lack of stress-induced c-fos expression in primary sensory processing areas of the rat brain. Neuroscience.

[56] Brooks B.A., Fahey T.D., Baldwin K.M. (2005). Exercise Physiology: Human Bioenergetics and Its Applications.

[57] Cambridge D., Whiting M.V. (1986). Evaluation of the qa interval as an index of cardiac contractility in anaesthetised dogs: Responses to changes in cardiac loading and heart rate. Cardiovasc. Res..

[58] Chang C.C., Hwang J.S., Chan C.C., Wang P.Y., Hu T.H., Cheng T.J. (2004). Effects of concentrated ambient particles on heart rate, blood pressure, and cardiac contractility in spontaneously hypertensive rats. Inhal. Toxicol..

[59] Light K.C., Dolan C.A., Davis M.R., Sherwood A. (1992). Cardiovascular responses to an active coping challenge as predictors of blood pressure patterns 10 to 15 years later. Psychosom. Med..

[60] Thomas G.D. (2011). Neural control of the circulation. Adv. Physiol. Educ..

[61] Irigoyen M.C., Krieger E.M. (1998). Baroreflex control of sympathetic activity in experimental hypertension. Brazilian J. Med. Biol. Res..

[62] Conti L.H., Shannon M.H., Murry J.D., Printz M.P. (2001). Repeated restraint stress-induced increase in baroreceptor reflex sensitivity: Role of corticotropin-releasing factor. Neuropeptides.

[63] Foster D.O., Frydman M.L. (1978). Nonshivering thermogenesis in the rat. Ii. Measurements of blood flow with microspheres point to brown adipose tissue as the dominant site of the calorigenesis induced by noradrenaline. Can. J. Physiol. Pharmacol..

[64] Marks A., Vianna D.M., Carrive P. (2009). Nonshivering thermogenesis without interscapular brown adipose tissue involvement during conditioned fear in the rat. Am. J. Physiol. Regul., Integr. Comp. Physiol..

[65] Takahashi J.S., Hong H.K., Ko C.H., McDearmon E.L. (2008). The genetics of mammalian circadian order and disorder: Implications for physiology and disease. Nat. Revi. Genet..

[66] Muller J.E., Stone P.H., Turi Z.G., Rutherford J.D., Czeisler C.A., Parker C., Poole W.K., Passamani E., Roberts R., Robertson T. (1985). Circadian variation in the frequency of onset of acute myocardial infarction. N. Engl. J. Med..

[67] Arntz H.R., Willich S.N., Oeff M., Bruggemann T., Stern R., Heinzmann A., Matenaer B., Schroder R. (1993). Circadian variation of sudden cardiac death reflects age-related variability in ventricular fibrillation. Circulation.

[68] Sheward W.J., Naylor E., Knowles-Barley S., Armstrong J.D., Brooker G.A., Seckl J.R., Turek F.W., Holmes M.C., Zee P.C., Harmar A.J. (2010). Circadian control of mouse heart rate and blood pressure by the suprachiasmatic nuclei: Behavioral effects are more significant than direct outputs. PLoS One.

[69] Vianna D.M., Allen C., Carrive P. (2008). Cardiovascular and behavioral responses to conditioned fear after medullary raphe neuronal blockade. Neuroscience.

[70] Rudy J.W., Huff N.C., Matus-Amat P. (2004). Understanding contextual fear conditioning: Insights from a two-process model. Neurosci. Biobehav. Rev..

[71] Morris R.W., Westbrook R.F., Killcross A.S. (2005). Reinstatement of extinguished fear by beta-adrenergic arousal elicited by a conditioned context. Behav. Neurosci..

[72] Dirikx T., Beckers T., Muyls C., Eelen P., Vansteenwegen D., Hermans D., D'Hooge R. (2007). Differential acquisition, extinction, and reinstatement of conditioned suppression in mice. Q. J. Exp. Psychol..

[73] Greenwood B.N., Strong P.V., Foley T.E., Fleshner M. (2009). A behavioral analysis of the impact of voluntary physical activity on hippocampus-dependent contextual conditioning. Hippocampus.

[74] Cordero M.I., Merino J.J., Sandi C. (1998). Correlational relationship between shock intensity and corticosterone secretion on the establishment and subsequent expression of contextual fear conditioning. Behav. Neurosci..

[75] Selye H. (1978). The Stress of Life.

[76] Campeau S., Nyhuis T.J., Sasse S.K., Kryskow E.M., Herlihy L., Masini C.V., Babb J.A., Greenwood B.N., Fleshner M., Day H.E. (2010). Hypothalamic pituitary adrenal axis responses to low-intensity stressors are reduced after voluntary wheel running in rats. J. Neuroendocrinol..

[77] Swenson R.M., Vogel W.H. (1983). Plasma catecholamine and corticosterone as well as brain catecholamine changes during coping in rats exposed to stressful footshock. Pharmacol. biochem. Behav..

[78] Bechtold A.G., Patel G., Hochhaus G., Scheuer D.A. (2009). Chronic blockade of hindbrain glucocorticoid receptors reduces blood pressure responses to novel stress and attenuates adaptation to repeated stress. Am. J. Physiol. Regul., Integr. Comp. Physiol..

